# AlGrow: A graphical interface for easy, fast, and accurate area and growth analysis of heterogeneously colored targets

**DOI:** 10.1093/plphys/kiae577

**Published:** 2024-11-05

**Authors:** Marcus McHale, Ronan Sulpice

**Affiliations:** Plant Systems Biology Lab, School of Chemical and Biological Sciences, Ryan Institute & Marei Centre, University of Galway, Galway, H91DK59, Ireland; Plant Systems Biology Lab, School of Chemical and Biological Sciences, Ryan Institute & Marei Centre, University of Galway, Galway, H91DK59, Ireland

## Abstract

AlGrow software provides a graphical interface to define target color volumes as hulls in color space and applies them to image segmentation and growth rate analysis across a multiplexed image series.

Dear Editor,

We previously reported a platform for image capture and analysis of multiplexed arrays of macroalgal lamina discs and applied this to an assessment of sea lettuce (*Ulva* spp.) growth diversity (Fort et al. 2019). In continued application to sea lettuce, and extension to other macroalgal species such as dulse (*Palmaria palmata*), we encountered issues segmenting images with poor color boundaries and more varied color distributions. Here, we report an approach that addresses these issues; the specification of a target color volume as a hull in color space with thresholding on color-distance to this hull surface. We provide AlGrow, a graphical interface software tool to calibrate and automate annotation and segmentation using the target-hull thresholding method (Fig. 1). AlGrow is suited to generating ground-truth labeled image masks for machine-learning, or as a standalone tool for growth rate analysis across an image series in plant and macroalgal phenotyping (Fig. 2).

**Figure 1.**
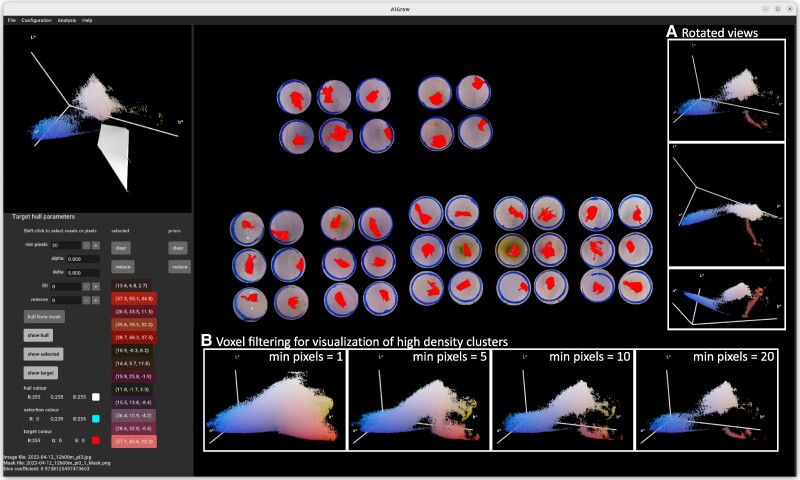
Target-hull specification and image segmentation on an image of *Palmaria palmata* (Supplementary File 1). Following prior layout detection and background masking (Supplementary Figs. S1 and S2), the loaded image is displayed alongside voxels representing pixel colors in the CIELAB color space (top-left window, see inset A for examples of rotated viewing angles). Filtering voxels by the number of pixels that each represents (“min pixels” = 20, see inset B for other values) allows visualization of higher density clusters. Voxels may be selected/deselected either from the 3D plot or by clicking the corresponding pixels in the image. Selected voxels are highlighted as spheres in the 3D plot and corresponding pixels are optionally overlaid in a custom color (“show selected” and “selection colour”). When a valid hull is constructed from selected points using the provided alpha parameter (“alpha” = 0 specifies no edge length limit, i.e. the convex hull), it is optionally displayed in the 3D plot (“show hull” and “hull color”). An optional overlay (“show target” and “target colour”) highlights pixels, whose corresponding voxel is either within the hull or within the supplied ΔE (“delta” = 5) of its surface. Selected voxels are represented by a button labeled with their coordinates in the “selected” column, where they may be removed individually, cleared, or reduced to a set consisting of only the current hull vertices. To support target-hull design across a series of images, when a new image is loaded, coordinates of the current selected voxels are copied to the list of “priors”, which are also considered as vertices during hull construction. Similarly, coordinates loaded from a configuration file or command-line arguments are considered as priors. For comparison across methodologies, a target mask may be loaded and the Dice coefficient is reported (0.97, bottom left). Loaded masks may also be used to automate hull construction, where the current min-pixels value and alpha parameter are considered during hull construction (“hull from mask”). The displayed convex hull was generated with less than 1 min of user interaction, while the loaded mask (supplementary file: “palmaria_alpha_mask.png”) is from a more refined alpha hull generated with approximately 10 min of user interaction across multiple images (Supplementary Fig. S3). Fill and remove are not used in this figure to provide a clear demonstration of the accuracy of target-hull segmentation; however, these functions are useful to account for local variation and/or materials that interfere with imaging, such as the nylon mesh in the macroalgal phenotyping apparatus. Configuration for this figure, including the selected hull vertices, is provided in Supplementary File 2.

**Figure 2.**
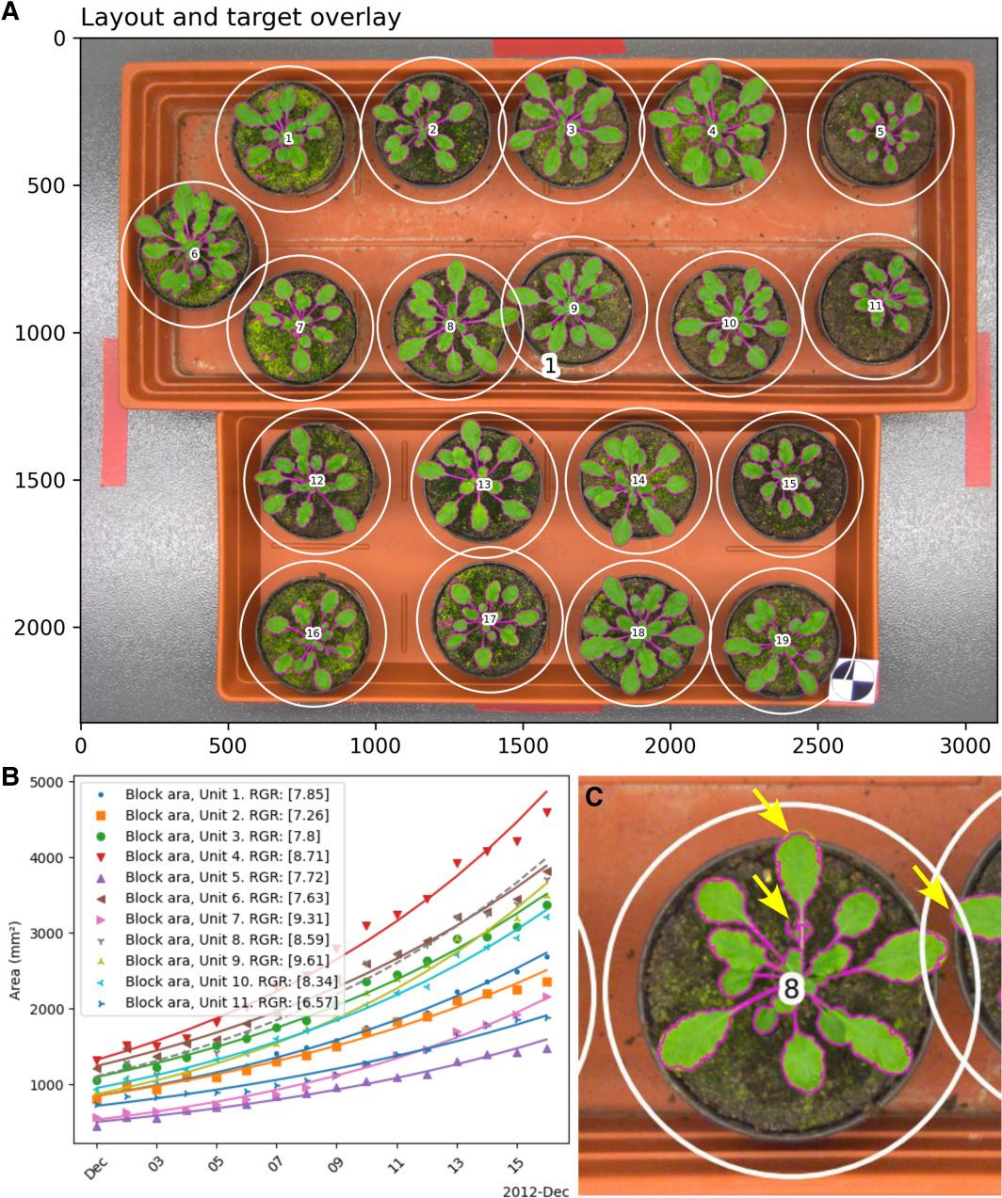
Image segmentation and growth rate analysis on Arabidopsis using the Ara2012 tray dataset obtained from http://www.plant-phenotyping.org/datasets (Minervini et al. 2016). A hull trained using only the first and last images of the 16 image Ara2012 series achieved Dice coefficients of similarity to the manually curated foreground masks of 0.969 ± 0.003 (mean ± standard deviation) for the remaining unseen images (Supplementary Table S1). **A)** Overlays are produced as part of the image debugging output from area analysis; presented is the overlay for the last image in the 16 image series. Overlays depict regions of interest from automated layout detection as white circles labeled with integer indices and segmentation boundaries are highlighted in magenta. **B)** For each unit, relative growth rate (RGR) is calculated as the slope of a straight line fit to the natural log of area over time. For each group, a plot is generated to display this line transformed back to units of area. Here, growth rate analysis was performed grouping units according to tray (Supplementary File 5). The dashed line for unit 8 indicates that the ModelFitOutlier flag was applied, suggesting a poor fit to the log-growth model and prompting further investigation. **C)** Inspection of unit 8 in the overlay images (here, image 12/16) identified potential issues indicated by arrows; vegetative damage that resembles herbivory by fungus gnat larvae, overlapping regions of interest, and a need to extend the hull to include colors of pixels representing leaf tips, particularly where they overlap the lighter colored tray. Configuration for segmentation performed for this figure is described in Supplementary File 6.

Manual region-of-interest specification and color space thresholding using the ImageJ graphical interface in our earlier pipeline were time-consuming, susceptible to operator bias and restricted by the need to load an image stack into memory (Schneider et al. 2012). Complex images required iterative selections as thresholds implicitly define cuboid boundaries in a 3-dimensional (3D) color space, which may be poorly aligned to the optimal decision boundary. We considered the intuitive selection of a set of target colors from the source image with a threshold applied to color-distance. However, this approach is similarly limited to spheroid geometries and, while complex selections require large sets of targets with a small radius, existing tools support less than 10 (Pethybridge and Nelson 2015; Merchuk-Ovnat et al. 2019). Unsupervised clustering of pixel colors is typically only suitable in simple imaging contexts, such as on a white background (Adams et al. 2020). Similar clustering in a hyper-dimensional color space with dimensional transformations can improve this approach, though such transformations impede re-use of the resulting selections (Henke et al. 2021). Further, the chosen K-means algorithm is reported to take ∼1 min for a single 5 megapixel (MP) image and has poor performance where clusters are of differing sizes, densities, and shapes. We excluded strategies that consider local neighborhood, topology, or shape information, preferring pixel-wise segmentation due to computational advantages and improved adaptability to varying morphologies and/or textures typical of many plant and macroalgal species. We also excluded supervised learning strategies as these depend on prior labeled data for training and may be computationally demanding.

AlGrow first down-samples pixel colors into representative voxels in the CIELAB color space (Fig. 1A; Supplementary Files 1 and 2) and provides a filter to visualize high density clusters (Fig. 1B). CIELAB is well suited to inspection of color distributions, being designed such that Euclidean distance in this space (ΔE/Delta E) is approximately proportional to perceived color difference. Color boundaries are defined by a hull in this space, with vertices selected by clicking on either voxel or pixel (Dawson-Haggerty 2019). Concave hull surfaces and/or disjoint color volumes can be defined by supplying the alpha parameter to limit edge length (Supplementary Fig. S1, Supplementary File 3) (Bellock 2015). Such alpha-hulls have previously been applied to color trait comparisons in ecological studies (Gruson 2020). In AlGrow, we have extended their application to image segmentation by applying a threshold to color differences, i.e. ΔE from the hull surface. A target hull may be generalized by selecting vertices across multiple images and saved to a configuration file for application across larger image sets (Weisburd 2023). To compare segmentation across different configurations or tools, a foreground mask is generated by AlGrow and may be loaded to calculate the Dice similarity coefficient (Fig. 2 and Supplementary Fig. S3, bottom left, Supplementary File 4).

Regions of interest may be detected in AlGrow using internal circular markers (Supplementary Fig. S2) (Xie and Ji 2002). Circle centers are then clustered to identify groups and apply relative indexing to rows or columns (Supplementary Fig. S3) (Virtanen et al. 2020). This is effective in the detection of retaining rings in the macroalgal phenotyping apparatus and for circular plant pots, as demonstrated calculating growth rates from the Ara2012 dataset (Fig. 2, Supplementary Files 5 and 6) obtained from https://www.plant-phenotyping.org/datasets-home (Minervini et al. 2016). A hull defined using the first and last images in this series was accurate across unseen images (0.969 ± 0.003, mean Dice ± SD, Supplementary Table S1). We applied this hull to the A1 test subset of Ara2012 that was previously evaluated across multiple learning methods, achieving comparable accuracy (0.968 ± 0.009 cf. 0.970 ± 0.008) with reduced processing time (257 ± 64 ms/image on a 3 GHz processor cf. 1.6 s/image on a 3.4 GHz processor) (Pape and Klukas 2015; Scharr et al. 2016).

User-guided specification of target color volumes as a hull with segmentation by surface distance thresholding is intuitive and predictably applied across an image series. The approach is computationally efficient, with segmentation of 8 MP images taking <5 s on a 3 GHz processor. A few additional seconds are required to generate debugging images, and optional layout detection typically takes 10 to 30 s. Array processing on specialized graphics or tensor processing units is not performed here but offers potential to further accelerate hull distance calculations, the most intensive operation in segmentation. Although not tested, hull distance has other potential applications, such as calculating vegetation indices in remote sensing.

AlGrow is developed in Python using established libraries (scipy, pandas, scikit-image, numpy) and is suitable for semi-automated labeling for machine-learning, or as a standalone application for area and growth analysis (McKinney 2010; Van der Walt et al. 2014; Harris et al. 2020; Virtanen et al. 2020). It is provided with a cross-platform graphical user interface with efficient 3D-rendering and distance calculations using Open3D (Zhou et al. 2018). Debugging figures for parameter tuning and summary reports are generated using matplotlib (Hunter 2007). AlGrow is free and released open-source with compiled binaries for major platforms, making it suitable for a broad community of users. These releases, a detailed guide and video tutorial explaining use of the software and its features, are all available on the project page, https://github.com/marcusmchale/algrow. We expect that this easy, fast, and accurate image analysis tool will be useful to the plant science community.

## Supplementary Material

kiae577_Supplementary_Data

## Data Availability

Software for this article is available in GitHub, at https://github.com/marcusmchale/algrow. Arabidopsis images used in this article are publicly available, at https://www.plant-phenotyping.org/datasets. All other data underlying this article are available in the article and in its online supplementary material.

## Supplementary data

The following materials are available in the online version of this article.

**Supplementary Figure S1.** Alpha hull target specification on Supplementary File 1 (“palmaria.jpg”, also used in Fig. 1) with fill and remove settings applied to create an accurate image mask.

**Supplementary Figure S2.** Circle detection is performed on an image constructed from the distance (ΔE) of each pixel color to a single color chosen to represent internal circular markers.

**Supplementary Figure S3.** Layout detection encompasses circle detection (Supplementary Fig. S2), clustering, sorting, and the application of indices to each region of interest.

**Supplementary Table S1.** Dice coefficient, accuracy, sensitivity, and specificity for each image in the Ara2012 and A1 test datasets, with additional processing times for A1.

**Supplementary File 1.** Image of *Palmaria palmata* used for Fig. 1 and Supplementary Figs. S1 to S3.

**Supplementary File 2.** AlGrow configuration file used for Fig. 1.

**Supplementary File 3.** AlGrow configuration file used for Supplementary Fig. S1.

**Supplementary File 4.** Foreground mask used for Fig. 1 and Supplementary Fig. S1.

**Supplementary File 5.** AlGrow sample details file used for Fig. 2.

**Supplementary File 6.** AlGrow configuration file used for Fig. 2.
