# Supplementary material for: AlGrow: A graphical interface for easy, fast, and accurate area and growth analysis of heterogeneously colored targets
Source: Plant Physiol. 2024 Nov 5;197(1):kiae577. doi: 10.1093/plphys/kiae577 (PMC11663580; doi:10.1093/plphys/kiae577)
Supplement: kiae577_Supplementary_Data [file kiae577_supplementary_data.zip › Supplementary Data.pdf]

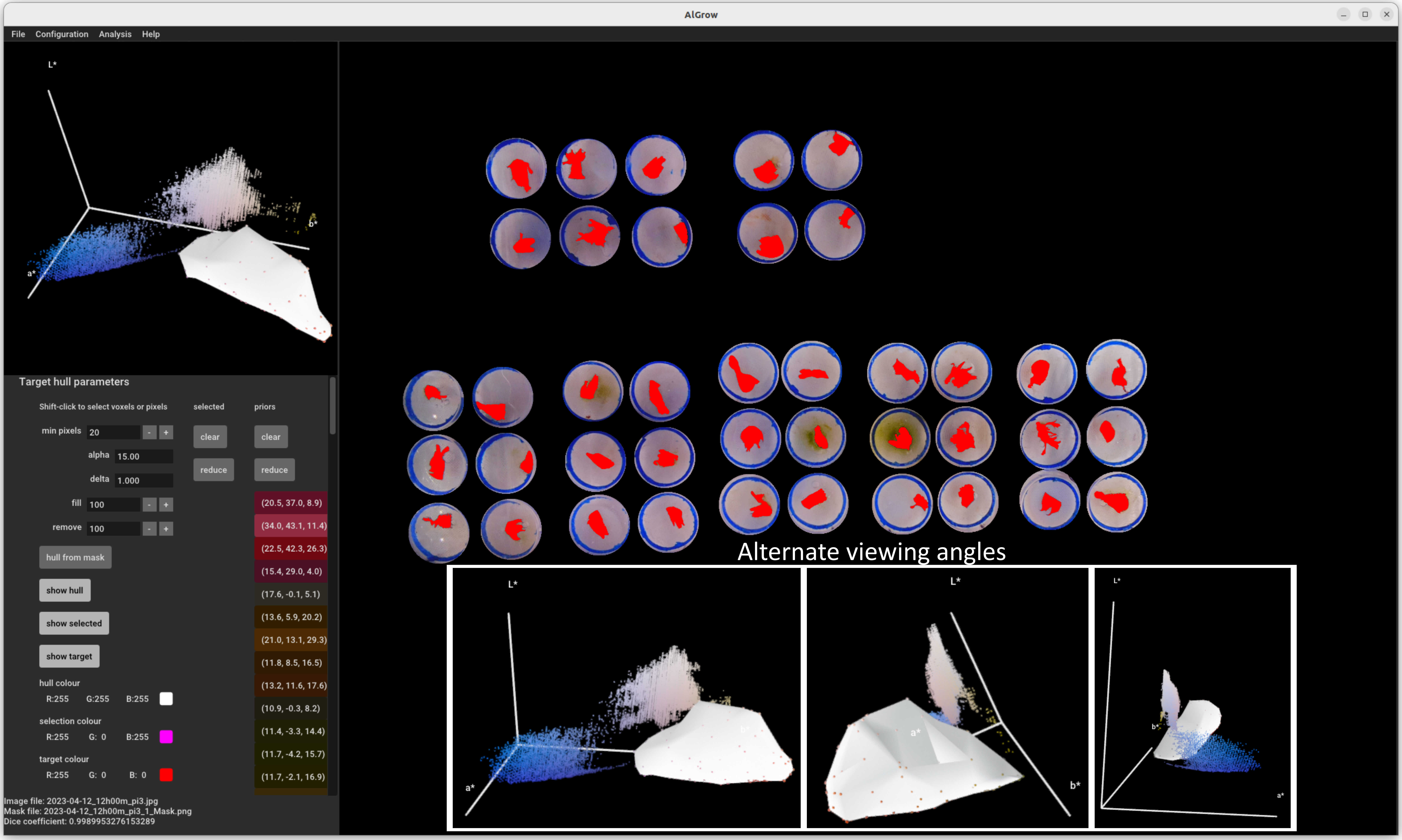

Figure S1. Alpha hull target specification on Supplementary File 1 ("palmaria.jpg", also used in Fig. 1) with fill and remove settings applied to create an accurate image mask. The Dice coefficient with the mask generated using this configuration is approximately but not exactly 1.0 due to voxel down-sampling applied in the GUI but not during formal area analysis. Alternate viewing angles are presented (inset) to best depict the concave surfaces of this hull. Configuration loaded into AlGrow for this figure is described in Supplementary File 3 (“palmaria_alpha.conf”).

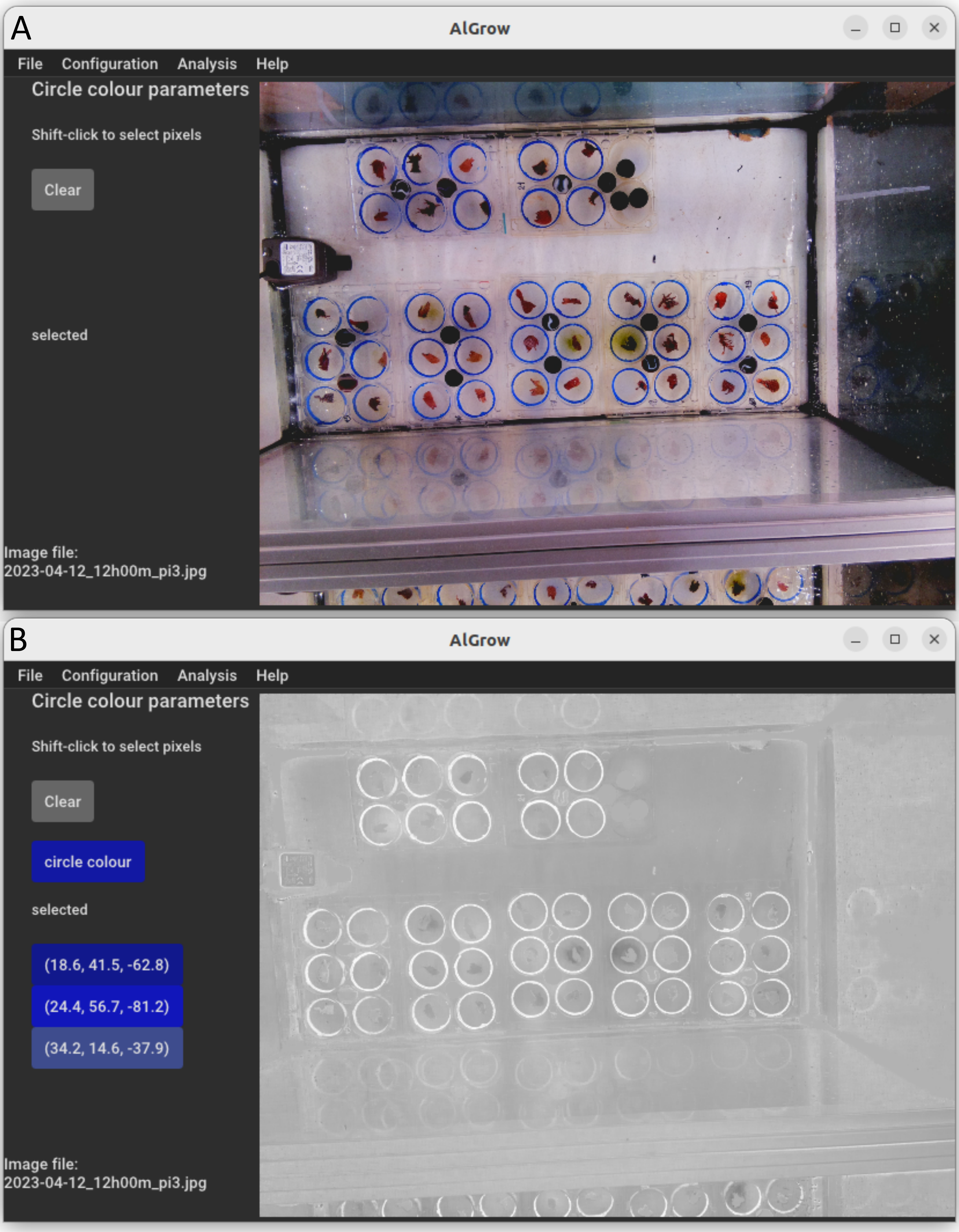

Figure S2. Circle detection is performed on an image constructed from the distance (ΔE) of each pixel color to a single color chosen to represent internal circular markers. The more complex procedure for hull construction (described in Fig. 1 and applied to image segmentation) is not applied here as the gradient from a single reference color is typically sufficient for edge detection. This figure depicts circle detection on Supplementary File 1 ("palmaria.jpg", also used in Fig. 1). A: A loaded image is displayed (right) and pixel colors may be selected. B: Selected colors are displayed as buttons (bottom left) with labels corresponding to CIELAB coordinates. Each selected color may be removed by clicking on the corresponding button, or all selected colors may be removed by clicking "Clear". The median of the selected color values in CIELAB, the current "circle colour", is displayed as a button below "Clear". The distance to this color is used to prepare the distance image for Canny edge detection (displayed at right).

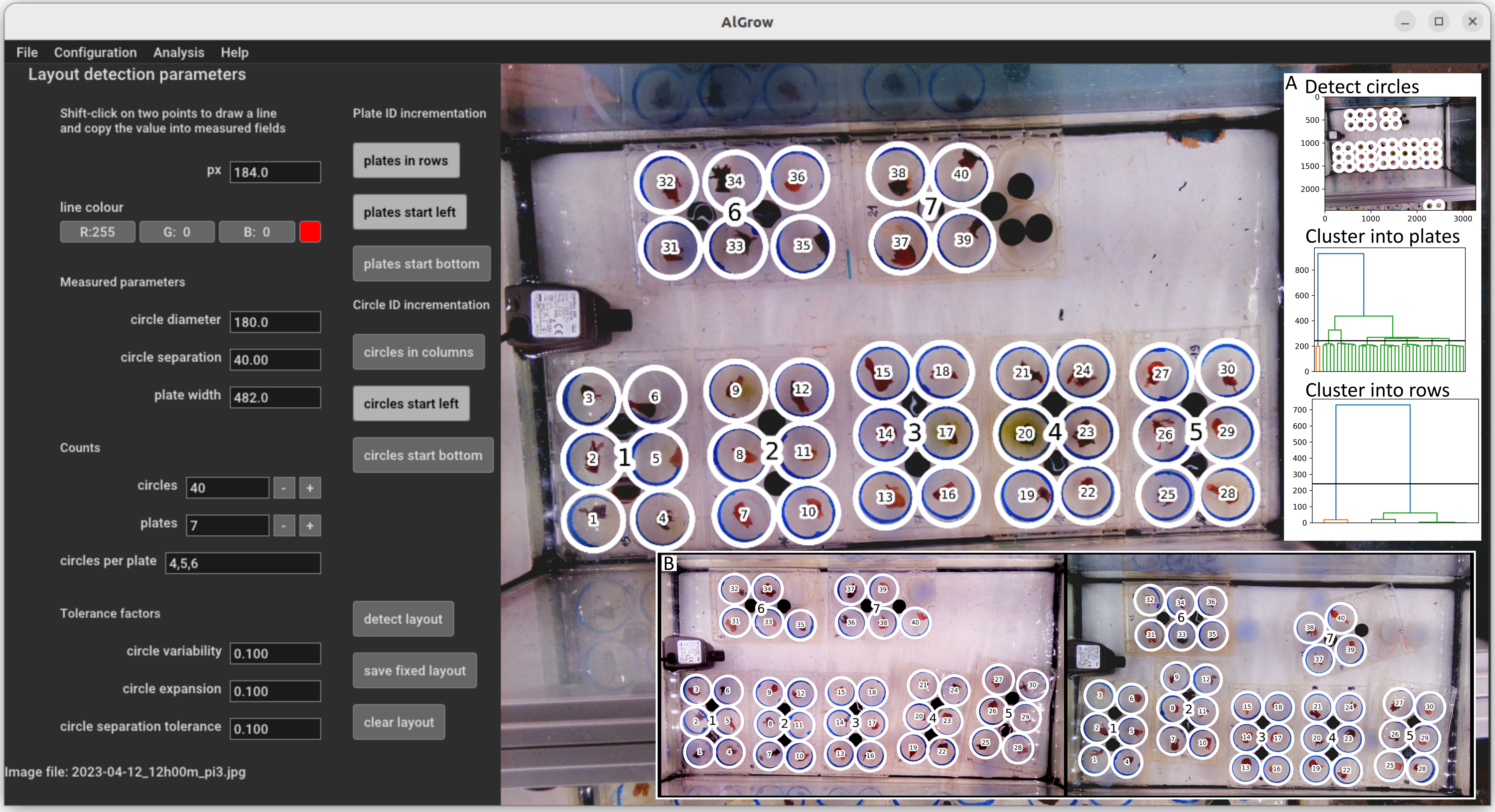

Figure. S3. Layout detection encompasses circle detection (Fig. S2), clustering, sorting and the application of indices to each region of interest. This figure depicts layout detection on Supplementary File 1 ("palmaria.jpg", also used in Fig. 1). A line may be drawn over the loaded image and the span is reported in pixels (px). This is used to establish circle diameter, circle separation (the distance between circle edges within a plate) and a plate width. Counts for the number of circles to detect in the image, the number of plates (clusters of adjacent circles) and the number of circles per plate (multiple options as a comma separated list) are also required. Tolerance factors may be supplied to affect circle variability (the range of circle diameters to consider), circle expansion for region of interest specification and circle separation tolerance for plate clustering. Incrementation options support alternative ordering of plate and circle IDs. When the "detect layout" button is clicked an attempt to identify the layout is made. Any errors in layout detection are reported with an appropriate debugging report, such as a cladogram where clustering does not meet the expectations. When layout detection is successful, an overlay is displayed detailing detected circular regions of interest and plates with corresponding IDs. **Inset A:** Following initial circle detection, clustering removes detected circles that are not within clusters of the prescribed 'circles per plate'. This automates the filtering of partially imaged plates. Plates are then clustered into rows (or columns) for annotation, and within each plate similar row or column clustering is applied to establish the order for ID incrementation. **Inset B:** A single layout specification may be robust to varying arrangements (left) and movement (right). Specifications may then be used to dynamically detect a layout in each image, or a fixed layout may be saved and later applied across all images where alternate arrangements and/or movement are not anticipated.
