# Supplementary figures and images for: AlGrow: A graphical interface for easy, fast, and accurate area and growth analysis of heterogeneously colored targets
Source: Plant Physiol. 2024 Nov 5;197(1):kiae577. doi: 10.1093/plphys/kiae577 (PMC11663580; doi:10.1093/plphys/kiae577)

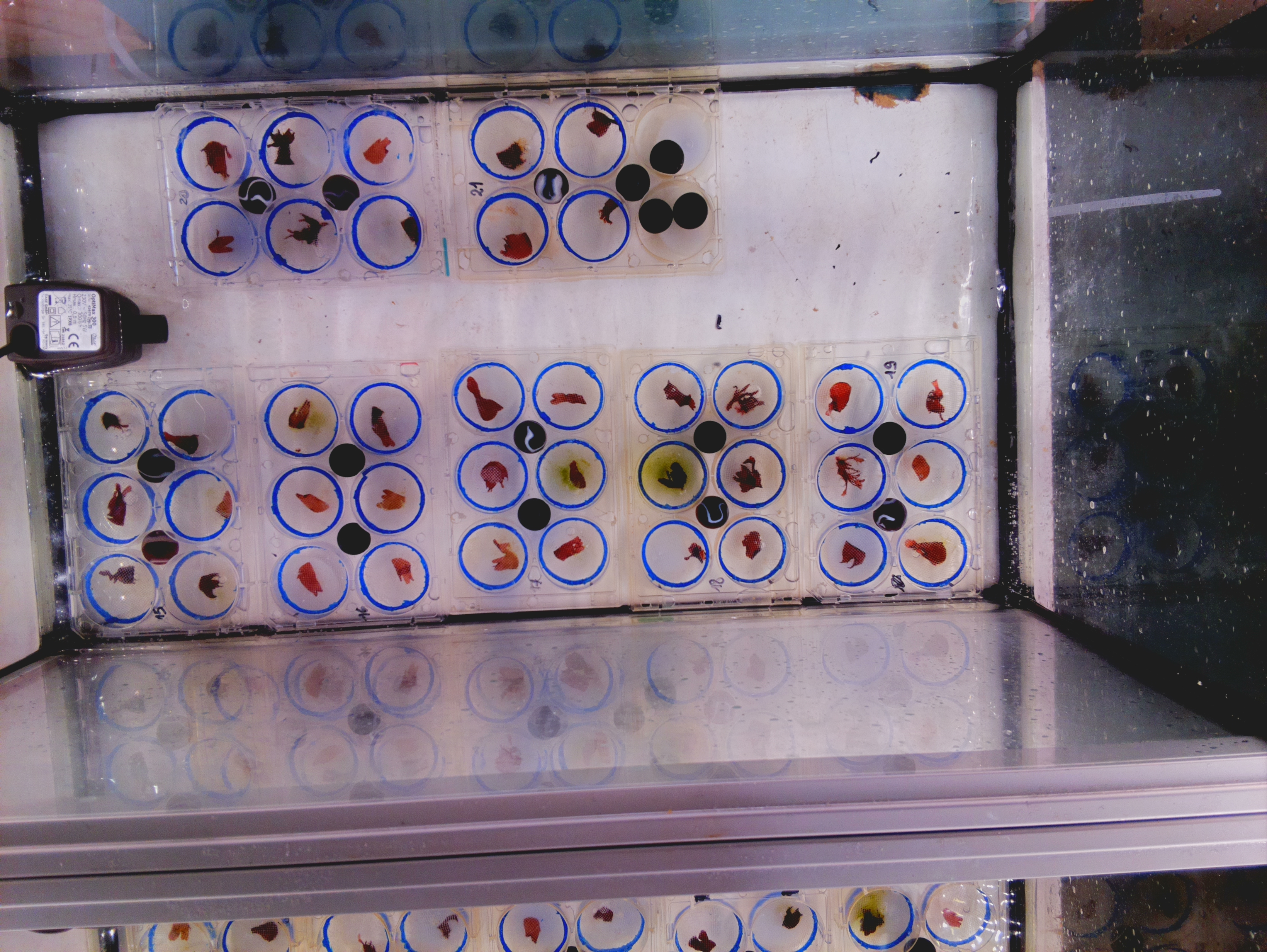

Supplement: kiae577_Supplementary_Data [file kiae577_supplementary_data.zip › palmaria.jpg]

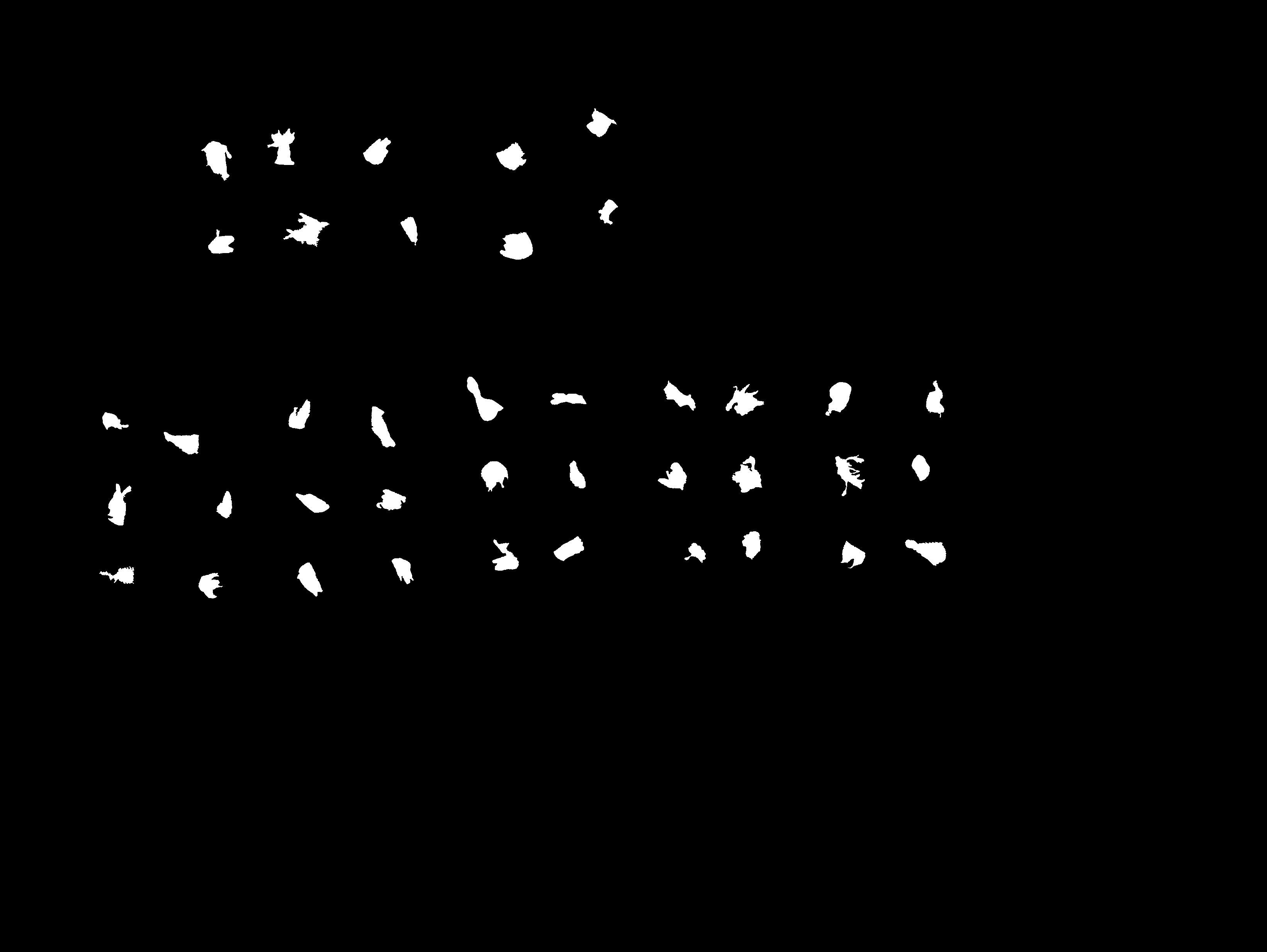

Supplement: kiae577_Supplementary_Data [file kiae577_supplementary_data.zip › palmaria_alpha_mask.png]
